# A novel fine-tuned deep-learning-based multi-class classifier for severity of paddy leaf diseases

**DOI:** 10.3389/fpls.2023.1234067

**Published:** 2023-09-05

**Authors:** Shweta Lamba, Vinay Kukreja, Junaid Rashid, Thippa Reddy Gadekallu, Jungeun Kim, Anupam Baliyan, Deepali Gupta, Shilpa Saini

**Affiliations:** ^1^ Chandigarh Engineering College, CGC Landran, Mohali, India; ^2^ Chitkara University Institute of Engineering and Technology, Chitkara University, Punjab, India; ^3^ Department of Data Science, Sejong University, Seoul, Republic of Korea; ^4^ Department of Research and Development, Zhongda Group, Jiaxing, Zhejiang, China; ^5^ Department of Electrical and Computer Engineering, Lebanese American University, Byblos, Lebanon; ^6^ School of Information Technology and Engineering, Vellore Institute of Technology, Vellore, India; ^7^ College of Information Science and Engineering, Jiaxing University, Jiaxing, China; ^8^ Division of Research and Development, Lovely Professional University, Phagwara, India; ^9^ Department of Software and CMPSI, Kongju National University, Cheonan, Republic of Korea; ^10^ Department of Computer Science and Engineering, Chandigarh University, Mohali, Punjab, India

**Keywords:** severity detection, multi-class classification, paddy diseases, severity classification, generative adversarial network

## Abstract

**Introduction:**

Paddy leaf diseases have a catastrophic influence on the quality and quantity of paddy grain production. The detection and identification of the intensity of various paddy infections are critical for high-quality crop production.

**Methods:**

In this paper, infections in paddy leaves are considered for the identification of illness severity. The dataset contains both primary and secondary data. The four online repositories used for secondary data resources are Mendeley, GitHub, Kaggle and UCI. The size of the dataset is 4,068 images. The dataset is first pre-processed using ImageDataGenerator. Then, a generative adversarial network (GAN) is used to increase the dataset size exponentially. The disease severity calculation for the infected leaf is performed using a number of segmentation methods. To determine paddy infection, a deep learning-based hybrid approach is proposed that combines the capabilities of a convolutional neural network (CNN) and support vector machine (SVM). The severity levels are determined with the assistance of a domain expert. Four degrees of disease severity (mild, moderate, severe, and profound) are considered.

**Results:**

Three infections are considered in the categorization of paddy leaf diseases: bacterial blight, blast, and leaf smut. The model predicted the paddy disease type and intensity with a 98.43% correctness rate. The loss rate is 41.25%.

**Discussion:**

The findings show that the proposed method is reliable and effective for identifying the four levels of severity of bacterial blight, blast, and leaf smut infections in paddy crops. The proposed model performed better than the existing CNN and SVM classification models.

## 1 Introduction

Agriculture is vital to boosting the economy of any nation. In India, agriculture alone makes up 18.8% of the gross domestic product (Economy Survey, 2021). Rice is a leading food crop and the most consumed agrarian product. Paddy is a primary source of sustenance for half the world’s population. Approximately 20% of the world population’s daily calorie demand is fulfilled by rice. Rice is cultivated almost everywhere; 10% of the world’s total agricultural land is used solely for the cultivation of the rice crop. This equates to 164.19 million hectares of land, of which 44 million hectares are found in India. Furthermore, 78% of the total rice production is directly used for human consumption, of which 90% is consumed in Asia only. Rice is traditionally the most substantial part of an Indian meal, and so a major portion of India’s cultivated land is used for rice cultivation. India is the second-largest producer of rice after China and the Indian economy is heavily reliant on rice production. A significant portion of total rice production is exported to other countries. A rapid growth in population increases the demand for agricultural products exponentially. Ultimately, this puts pressure on the agricultural industry to increase productivity. As agricultural land is limited, to increase production, work should be carried out to decrease losses in rice yield. Currently, approximately 20%–100% of rice crop yields are devastated by rice diseases (Dhiman et al., 2023).

Rice is a Kharif crop that performs best in warm, humid weather and flooded areas. This creates a favorable environment for a variety of diseases to thrive. Based on cause, diseases in rice crops can be divided into two groups. Diseases caused by biotic factors or an organism are parasitic. Parasites include pathogens, pests, and weeds. Pathogens, including viruses, bacteria, and fungi, can cause a wide range of diseases. Out of all these diseases, some of the rice diseases are more likely to take hold and severely affect the yield of the crop. Other factors that cause a reduction in the yield of a field are non-parasitic diseases. Non-parasitic diseases are caused by unfavorable temperatures, irradiation, deficiencies of specific nutrients, and water. Alkalinity, bronzing, cold injury, panicle blight, straighthead, and white tip are examples of abiotic diseases.

Diseases can also be classified based on the part of the crop affected. Symptoms of the disease can appear on the stem, panicle, sheath, or leaf (Dutta et al., 2023). All these diseases cause a loss in the yield of the crop. The magnitude of the reduction in yield is directly influenced by the level of severity of the disease. A disease has different levels of severity: mild, moderate, or severe. To increase the production of rice it is essential to prevent these diseases from occurring or to detect the disease and ascertain its severity level before it affects the yield of the crop. The identification or detection of disease requires careful and in-depth observation of different parts of the plant. Previously, it has been difficult to detect diseases as it requires manpower and expertise to identify the disease from the symptoms. However, the recent expansion of the application of computational approaches, facilitated by the rapid development of computer vision techniques, has meant that computer vision-based automation has become a popular method for diagnosing and monitoring plant diseases (Kashif et al., 2023) so that they can be cured before spreading across the whole plant and destroying the panicle. In practical terms, detecting a disease and classifying its severity helps farmers to prevent or cure the disease and determine the potential loss in yield of the crop.

### 1.1 Contribution

The contributions of this research are as follows:

- The creation of a deep-learning hybrid classifier that first locates the area afflicted by the disease, then categorizes the condition according to its severity level.
- The ability to identify and categorize the paddy plant’s infected region using the hybrid model that has been developed.
- A potent method for automatically determining the severity of the disease that may be improved to offer a uniform paddy plant disease diagnosis system for use in real-world scenarios.
- Support for early disease mitigation and prevention, and the potential to reduce disease costs while protecting the environment internationally.

### 1.2 Outline

A literature review of the research field of image cataloging is carried out in section 2. In section 3, the suggested novel approach is described, with a discussion of the algorithm used in the paper and a detailed description of the proposed crossbreed classifier. The results and research findings are presented in section 4. The anticipated model is equated with the existing classifier by performance measures. The hybrid model is also compared with the basic classification algorithms using the same dataset. The conclusion and future scope are discussed in section 5.

### 1.3 Objectives of the paper

The proposed fusion model’s primary goals are:

- To increase the dataset of images for the three paddy infections, blight, leaf smut, and paddy blast, using generative adversarial network (GAN) augmentation.
- To detect the three paddy infections and determine the disease severity level using segmentation techniques.
- To classify the paddy diseases based on the type of infection and disease severity level in the paddy.

## 2 Literature review

It is very difficult to categorize the intensity of paddy leaf disease. A wide range of studies has produced varied results. This section discusses several of these findings. Lamba, Baliyan, and Kukreja (Lamba et al., 2022a) proposed a novel hybrid classification model combining two popular classification approaches, a convolutional neural network (CNN) and a long short-term memory (LSTM) network. Classification is based on the type of rice leaf disease. The three rice leaf diseases considered in this article are bacterial blight, blast, and leaf smut. An overall accuracy of 97% was achieved by the hybrid model. The images were collected from both fields and online resources. After data collection, the dataset was augmented using a generative adversarial network (GAN). The authors (Lamba et al., 2022b) also investigated the effect of generative adversarial network augmentation on the CNN classification algorithm. For model testing and training, three paddy leaf infections were being considered. In predicting the disease, the classifier had a high accuracy of 98.23%. Patil and Kumar (2022) combined multi-layer perceptrons with the classification algorithm to classify three diseases. Using an image set of 3,200 images, the classifier achieved a correctness rate of 95.31%. Deng et al. (2021) used CNN to classify six different paddy diseases with 91% accuracy. The dataset contained 33,026 self-generated images of six diseases. The training precision was approximately 92% with a CNN architecture model. Priyangka and Kumara (2021) used VGG19, a CNN model, to categorize images according to seven different paddy diseases. The dataset comprised 105 images (15 images for each disease) from three diverse sources. Overall, the success rate was 95.4%. The authors used data extension to increase the size of the image set. Mekha and Teeyasuksaet (2021) used the random forest method and attained a prediction rate of 69%. The dataset contained 120 images of infected leaves. Luo et al. (2019) categorized four diverse paddy illnesses using a model combining CNN and support vector machine (SVM). Using a self-collected dataset of 6,637 images, the authors achieved 96.8% accuracy. Ghosal and Kamal (2020) used CNN to classify three rice ailments and achieved 92.46% accuracy. Goluguri et al. (2021) used CNN combined with SVM for feature extraction and prediction. There were 1,600 images in the dataset. The model had a 97.5% accuracy rate. Bhattacharyya and Mitra (2019) used CNN classification to predict three paddy diseases. The classifier achieved a correctness rate of 94%. There were 1,500 images in the dataset, i.e., 500 of each disease (Baroudi et al., 2021).

Dastider et al. (2021) used lung ultrasounds to classify the severity of COVID-19 illnesses using a CNN–LSTM hybrid model. The auto-encoder network with CNN and LSTM used in this study was proposed as a reliable and noise-free model. Maragheh et al. (2022) developed a hybrid approach for multi-label text classification by combining the most precise features of LSTM with a spotted hyena optimizer. The model was tested on four different datasets. This article also compared six other fusion approaches using LSTM to produce the best performance. She and Zhang (2018) employed the CNN–LSTM hybrid technique for text classification. The LSTM algorithm was used to store historical data. For text classification, Zhang et al. (2018) used an LSTM–CNN hybrid approach. Overall, the success rate was 91%. The classifier was tested against plain CNN models, LSTM simple models, and LSTM–CNN using various filter sizes. The best results were obtained using an LSTM–CNN hybrid model with a filter size of 5X 600 pixel. Tee et al. (2022) provide an overview of various action recognition strategies. The paper describes the mixed system created by combining CNN and LSTM and provides a brief summary of studies that used both strategies. Waldamichael et al. (2022) reviewed 45 publications on the diagnosis of plant disease, which included information on classification techniques, datasets, accuracy, and strategies. Dhiman and Vinod (2022) rused the CNN approach to identify and categorize paddy illnesses. The classifier divided the dataset into three categories: healthy, unhealthy but curable, and unhealthy and incurably intense. The dataset contained 650 sample photos. Hassan and Maji (2022) used the CNN algorithm to classify four paddy leaf diseases. The diseases under consideration were paddy blast, blight, tungro, and brown spots. Babu et al. (2022) used the CNN technique to classify four rice diseases (Adedoyin et al., 2022).

Saidi et al. (2020) employed a CNN–SVM combined approach to detect depression. The CNN–SVM cross-classifier produced a precision rate of 68% using the Distress Analysis Interview Corpus/Wizard-of-Oz (DAIC-WOZ) dataset. The database comprised a training set and evaluation set 2,480 and 560 bytes in size, respectively. In another study, the CNN–SVM integration was used by the researchers to recognize and catalog brain tumors using magnetic resonance imaging (MRI) images (Zhou et al., 2019). According to the experimental results, brain tumors can be categorized with 98.49% accuracy. The combined approach was also compared with other classification approaches. A new adaptive machine (Sun et al., 2017) has been suggested for the categorization of MRIs. The new proposal has an estimated accuracy of 99.5%. The authors used data from Haxby’s functional MRI dataset from 2001. The study by Ahlawat and Choudhary (2020) sought to distinguish between manually written digits. A composite model of CNN–SVM was applied to the Modified National Institute of Standards and Technology (MNIST) image set. This method had an accuracy of 99.28%. For analyzing hyperspectral images, Leng et al. (2016) used a CNN–SVM combined technique. In a study by Niu and Suen (2012), handwritten characters are recognized and classified. The MNIST dataset was utilized to train the classifier, and the digit classification accuracy was 99.81%. The studies reviewed are presented in **Table 1**, along with a summary of the cataloging methodology utilized. Jiang et al. (2020) applied a CNN–SVM mixed algorithm to 8,912 images of four paddy diseases with the aim of classifying the diseases. All conditions were labeled as leaf diseases. The CNN–SVM model achieved 96.84% accuracy. Hasan et al. (2019) used CNN and SVM to identify nine paddy crop diseases. A dataset of 1,080 infected leaf images was created. The model categorized the diseases with 97.5% accuracy. Liang et al. (2019) achieved a 99.2% accuracy rate for the detection of paddy blast disorder. The dataset included 3,010 images of healthy and diseased plant leaves (Upadhyay et al., 2022).

**Table 1:** Summary of the literature review.

Citation with year	Diseases considered	Model framework	Category	Accuracy of model (%)
Lamba et al., 2022a	3 rice leaf disease	CNN–LSTM with GAN	Disease classification	97
Lamba et al., 2022b	3 rice leaf diseases	GAN and CNN	Disease classification	98.23
Patil and Kumar, 2022	3 rice leaf diseases	CNN with IoT	Disease classification	95.31
Deng et al., 2021	6 rice leaf diseases	CNN	Disease classification	91
Priyangka and Kumara, 2021	7 rice leaf diseases	CNN model VGG19	Disease classification	95.4
Mekha and Teeyasuksaet, 2021	Rice leaf diseases	Random forest	Disease classification	69
Luo et al., 2019	4 rice leaf diseases	CNN with SVM	Disease classification	96.8
Ghosal and Kamal, 2020	3 rice leaf diseases	CNN transfer learning	Disease classification	92.46
Goluguri et al., 2021	Rice leaf diseases	DCNN with SVM	Disease classification	97.5
Bhattacharyya and Mitra, 2019	3 rice leaf diseases	CNN	Disease classification	94
Dastider et al., 2021	COVID-19	DCNN with SVM	Severity classification	97.5
She and Zhang, 2018	Text classification	CNN–LSTM	Image classification	90.68
Zhang et al., 2018	Text classification	LSTM–CNN	Image classification	91.17
Waldamichael et al., 2022	Cereal crop disease	CNN	Severity classification	89
Dhiman and Vinod, 2022	Rice disease	CNN	Disease classification	97.692
Hassan and Maji, 2022	Rice leaf diseases	CNN	Disease classification	99
Babu et al., 2022	Rice leaf diseases	CNN	Disease classification	99.45
Saidi et al., 2020	Depression	CNN–SVM hybrid	DAIC-WOZ	68
Khairandish et al., 2022	Brain tumor	CNN–SVM hybrid	BRATS	98.50
Sun et al., 2017	MRI	CNN–SVM hybrid	Haxby’s 2001 fMRI dataset	99.65
Ahlawat and Choudhary, 2020	Biotic stress in paddy	CNN–SVM hybrid	MNIST dataset	99.30
Niu and Suen, 2012	Skin lesion	CNN–SVM hybrid	MNIST dataset	99.18
Jiang et al., 2020	Paddy infections	CNN–SVM hybrid	Self-created	96.79
Hasan et al., 2019	Paddy infections	CNN–SVM hybrid	Self-created	97.49
Liang et al., 2019	Paddy blast infections	CNN–SVM hybrid	Self-created	99.19
Gulzar, 2023	Fruit classification	TL-MobileNetV2 model	Kaggle dataset	99
(Lamba et al., 2023)	Blast severity classification	CNN–SVM	Kaggle, GitHub, UCI	97
Lamba et al., 2022c	Rice disease classification	SVM	Kaggle, GitHub, UCI	96.23
Gulzar et al., 2020	Seed classification	CNN	Self-created	99
Mamat et al., 2023	Fruit classification	Transfer learning	Self-created	99.5

fMRI, functional MRI; IoT, internet of things; MNIST, Modified National Institute of Standards and Technology; UCI, University of California, Irvine, Machine Learning Repository; BRATS, Multimodal Brain Tumor Image Segmentation Benchmark; DCNN, Deep CNN; DAIC-WOZ, Distress Analysis Interview Corpus/Wizard-of-Oz set.

## 3 Materials and methods

A proposed hybrid model for detecting the diseased area and disease severity level of paddy leaves for three infectious diseases, bacterial blight, blast, and leaf smut, was constructed and consists of six modules. The first module is dataset preparation, which comprises dataset collection from both primary and secondary data sources and the distribution of the image set according to the type of disease. It targets the collection of images of paddy leaf infected by three paddy infections. The data is then pre-processed in module two. Pre-processing standardizes the pictures from numerous possessions in an identical format. The third module increases the size of the dataset exponentially using GAN amplification techniques. In the fourth module, disease severity is determined for every leaf in the image set using the segmentation technique. The disease severity level is also annotated on the image in module four. In the fifth module, a multi-class hybrid classification model is generated by combining the characteristics of CNN and SVM. In the last module, the hybrid classifier is trained and verified against the training set and test set individually.

### 3.1 Dataset preparation

Dataset preparation is the creation of an inventory of potential data sources with the categorized data required to feed the classification model. It entails gathering and disseminating data. Data collection is the process of exploring primary and secondary data sources to obtain the required data. Data from primary sources can be defined as data collected from surveys, observations, and experiments. These kinds of data are directly collected by the researcher. The data are original, raw data that need to be pre-processed before feeding into the model (Kour et al., 2022). This produces reliable, qualitative data but is a more costly data collection method.

Secondary data is collected by another person. This is a less costly data collection method, but the collected data are less reliable and authentic. The data can be taken from previously published sources or unpublished sources. The collected data are then divided based on the type of infection and a separate folder is created for each category of infection.

#### 3.1.1 Data collection

Diseases in rice have a significant impact on grain quality, market segments, and revenue. Classification processes using object recognition and deep learning require a good dataset. All images in the dataset were collected from both primary (self-collected) and online sources. Primary data were collected from a farm in Patiala, Punjab, India, from July 2021 to August 2022. The images were taken under sunlight. A mobile camera of 12 mega-pixels with a f/2.20 aperture and 1.250 micro-pixel size was used for data collection.

The size of the images captured from the primary source was 3,008 × 4,016 pixels. A total of 533 images were collected from the primary source: 202 showed bacterial blight infection, 218 showed leaf blast, and 113 images showed leaf smut-infected paddy leaves.

**Figure 1:**
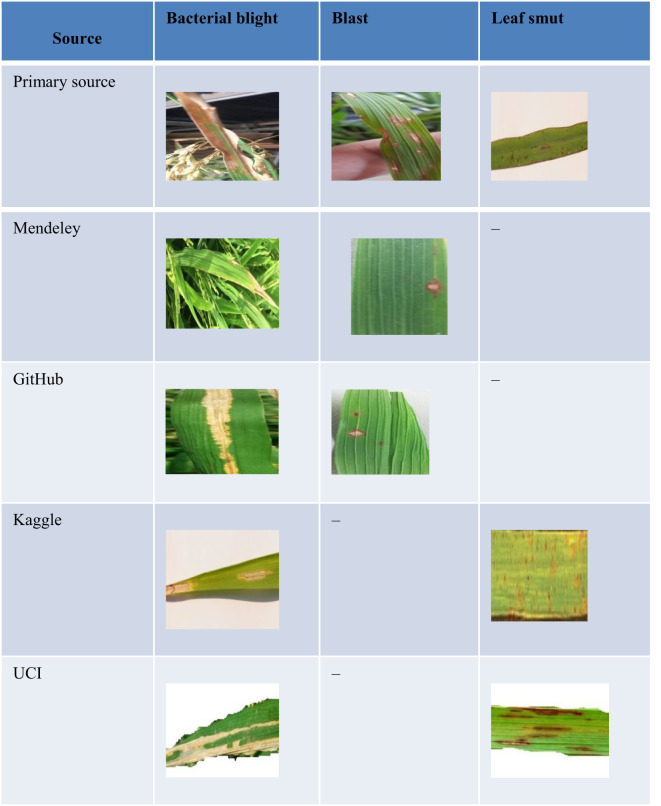
Sample pictures from the primary and secondary data resource.

Secondary data were gathered *via* the online repositories Mendeley, Kaggle, GitHub, and the University of California, Irvine (UCI), Machine Learning Repository. A total of 3024 images were taken from the Mendeley repository, comprising 1,584 and 1,440 pictures of blight and leaf blasts, respectively. In addition, 80 images each were taken from UCI and Kaggle: 40 images of bacterial blight and 40 pictures of leaf smut infection. Overall, 3,535 images were collected from secondary resources. The collected data were then separated into sets based on the type of paddy infection. **Figure 1** presents sample pictures from the primary and secondary data resources. There were certain limitations to the dataset thus collected. The calculation of disease severity based on the area of the affected leaf highly depends on the proportion of leaf visible in the image. This meant that if the image did not show the whole leaf then the calculation of disease severity level based on area was affected. The dataset was compiled from various sources; therefore, ensuring that the complete leaf appeared in every image would have been a time-consuming task. The attributes of the images were selected and extracted by the convolutional neural network.

#### 3.1.2 Data distribution

At this stage, the data collected from various sources were divided into groups based on the type of paddy infection. Separate folders are created for each paddy disease, and all the images of paddy leaves infected by that particular infection were placed in that folder. The dataset as a whole comprised 4,068 images: 2,058 images of bacterial blight-infected leaves, 1,817 images of leaf blast infection, and 193 images of leaf smut infection. **Table 2** presents detailed information on the number of images provided by each source and for each infection type.

**Table 2:** Number of images collected by source and infection type.

Rice leaf disease	Secondary resources				Primary resources	Total
	Mendeley	Kaggle	UCI	GitHub		
Bacterial blight	1,583	41	41	191	202	2,058
Leaf blast	1,440	0	0	159	218	1,817
Leaf smut	0	40	40	0	113	193
Total	3,024	80	80	351	533	4,068

#### 3.1.3 Data pre-processing

After taking the images from publicly available sources, the images were pre-processed to prepare them for obtaining the severity of the paddy diseases. All the images were taken from a variety of sources.

Each source used different equipment for data collection and hence the images from the different sources were of different dimensions. To feed the images from the dataset to the model it was essential to make the images identical in all forms. **Table 3** describes the images from the various sources in terms of the dimensions of the images (Deepa et al., 2020). To maintain the homogeneity of the image set in terms of the dimensions of the pictures, three pre-processing techniques were applied: standardization, normalization, and rescaling. Standardization is one of the most effective feature-scaling techniques. It is also known as Z-score normalization. Used when the feature distribution is normal or Gaussian, it compresses or expands data by transforming it into a mean vector of the source records.

**Table 3:** Dimensional information of images from each source.

Characteristic	Primary source	Secondary source			
		Mendeley	GitHub	Kaggle	UCI
Plant type	Rice	Rice	Rice	Rice	Rice
Number of different diseases	3	2	2	2	2
Image width	1,908 pixels	300 pixels	300 pixels	756 pixels	756 pixels
Image height	4,032 pixels	300 pixels	300 pixels	250 pixels	250 pixels

**Figure 2:**
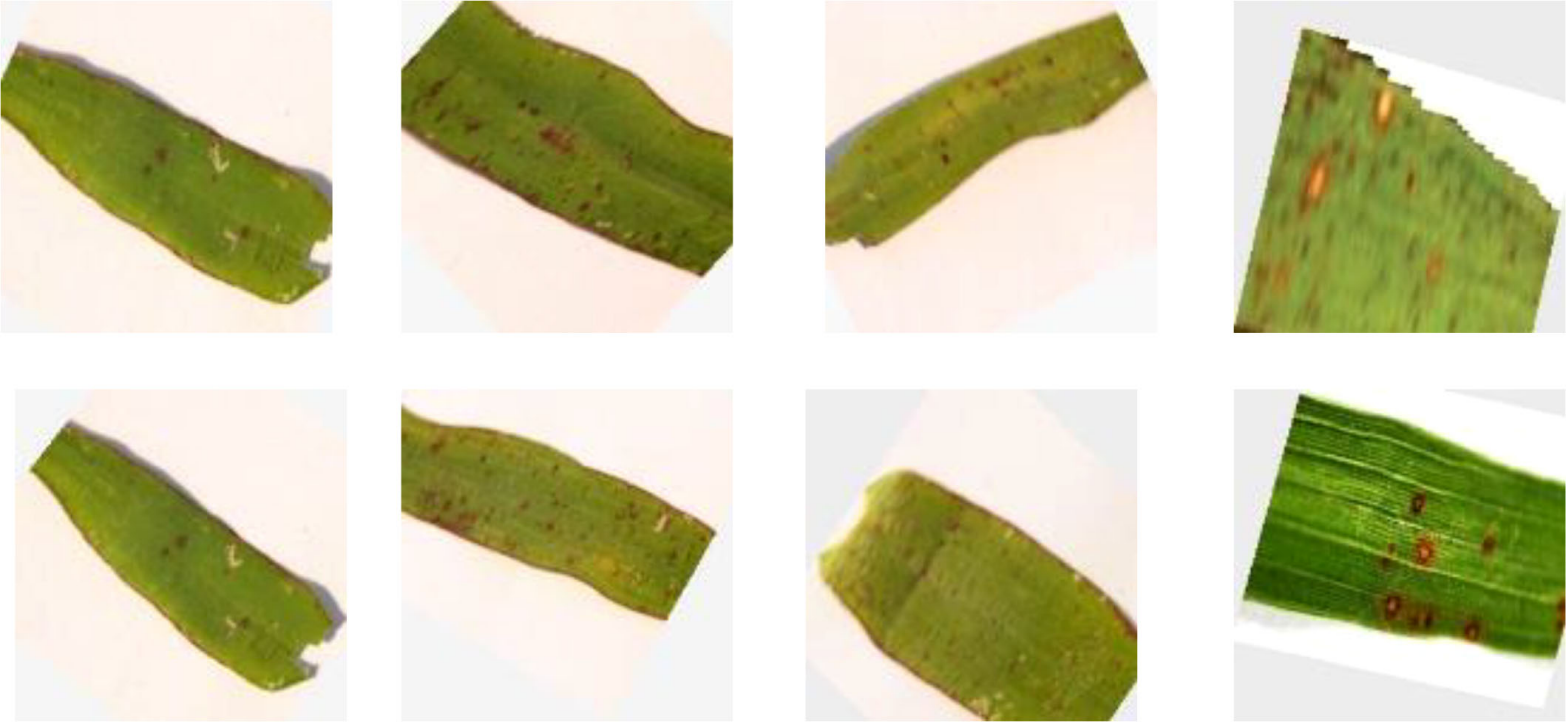
Sample images after pre-processing.

Normalization is also known as min–max scaling. It is used to transform topographies to the same scale. This scale ranges between 0 and 1. In rescaling, the dimensions of the images are changed to form a uniform dataset. In this paper, ImageDataGenerator from the Keras library was used for the three pre-processing activities applied to the images of the dataset. After pre-processing, the dataset comprised identical images in terms of dimensions. ImageDataGenerator can also be used for image augmentation. **Figure 2** shows a sample of the images after pre-processing of the dataset.

### 3.2 Data augmentation using GAN

**Figure 3:**
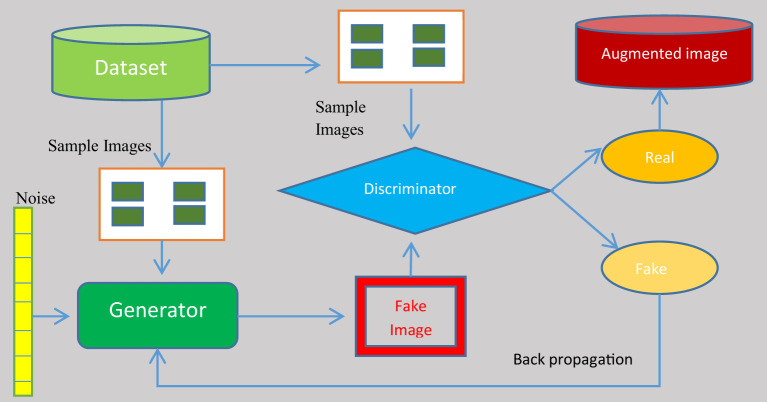
Structure of the generative adversarial network.

To eliminate the over-fitting of the anticipated system, the records generated were augmented with images from the dataset. A GAN was used to augment the image set. The deep-learning model known as a generative adversarial network (GAN) pits two neural networks against one another in the context of a zero-sum game. GANs are designed to produce new, synthetic data that closely mimic a pre-existing data distribution. GAN is employed to generate new photos that are identical to the original images. It can be utilized immediately in model training. Its architecture makes use of two neural networks: a generator and a discriminator. The generator’s objective is to produce a fictional output. It incorporates random noise and generates output that is as near as possible to the actual signal. To discriminate between fake and real images, the discriminator is fed fake images from the generator. In addition, it gives the generator feedback on its effectiveness. Based on this feedback, the generator adjusts its methodology in the following iteration to generate outcomes that are more realistic. As time goes on, its productivity improves. The discriminator finally reaches a point where it is unable to distinguish between genuine and fake images. **Figure 3** demonstrates the structure of the working GAN network.

A generator and a discriminator are both present in a GAN. The generator attempts to trick the discriminator by creating fake samples of data (such as an image, audio, etc.). On the other hand, the discriminator tries to tell the difference between the genuine and fraudulent samples. Both the generator and the discriminator are neural networks, and throughout the training phase they compete with one another. The procedures are repeated multiple times, and, each time, the generator and discriminator improve. The challenge encountered in GAN augmentation is that the images generated are slightly different from the genuine images. There is very small difference in the features of the images generated so severity of the diseases in the images taken from a sample images is more or less equal.

The discriminator’s goal is to correctly label the picture produced by the generator as false while correctly labeling the original images as true. The discriminator’s loss function is:


(1) $$\text{Los}{\text{sD}}_{\;}{=}_{\;}{\text{f}}_{\text{Difference}}\left(\text{D}\left({\text{I}}_{\text{Real}}\right),1\right)\;+\;{\text{f}}_{\text{Difference}}\left(\text{D}\left(\text{G}\left({\text{I}}_{\text{Fake}}\right)\right),0\right)$$

The loss of the discriminator is calculated by adding the two functions and subtracting the functional parameters. The discriminator’s focus is to reduce the loss. The discriminator’s assessment of a true image is compared with 1 in the initial operant of the formula and to 0 in the second. The formula can alternatively be expressed as follows:


(2) $$\text{Los}{\text{sd}}_{\;}{=}_{\;}{\text{f}}_{\text{Max}}\left\{\text{log}(\text{D}\left({\text{I}}_{\text{Real}}\right)\;+\text{ log}\left(1-\text{D}\left(\text{G}\left({\text{I}}_{\text{Fake}}\right)\right)\right)\right\}$$

The generator’s goal is to confuse the discriminator as much as possible, such that the resulting picture is labeled as true. The following equation describes the generator’s loss function:


(3) $$\text{Los}{\text{sG}}_{\;}{=}_{\;}{\text{f}}_{\text{Difference}}\left(\text{D}\left(\text{G}\left({\text{I}}_{\text{Fake}}\right)\right),1\right)$$

By multiplying the variation in the function of the set of parameters by the discriminator’s judgment value of the fake image, 1, the loss of the generator is calculated. The following is another way to present the loss function:


(4) $$\text{Los}{\text{sG}}_{\;}{=}_{\;}{\text{f}}_{\text{Min}}\left(\text{log}\left(\text{D}\left(\text{G}\left({\text{I}}_{\text{Fake}}\right)\right)\right)\right)$$

Equation 5 represents the whole loss function for the GAN model. The generator’s objective is to reduce the function, while the discriminator’s objective is to maximize the function:


(5) $$\text{Los}{\text{s}}_{\text{GAN}}{=}_{\;}\text{mi}{\text{nG}}_{\;}\text{ma}\;{\text{xD}}_{\;}\left\{\text{log}(\text{D}\left({\text{I}}_{\text{Real}}\right)\;+\text{ log}\left(1-\text{D}\left(\text{G}\left({\text{I}}_{\text{Fake}}\right)\right)\right)\right\}$$

The GAN augmentation increases the size of the dataset enormously. After GAN, the dataset of 4,068 images increased to a dataset of 9,175 images. Augmentation increased the images of blight, blast, and leaf smut by 424%, 180%, and 922%, respectively. The images generated through GAN were of high quality and the best match to the category of the samples are taken by the GAN to generate new image.

The collected information was then divided according to a ratio of 80:20 into a training dataset and a test dataset. The training set was further divided according to an 80:20 ratio into a training dataset and a validation dataset. The hybrid model was trained on the training dataset in order to classify the paddy disease according to both type and severity. The testing dataset evaluated the effectiveness of the proposed classification model, whereas the validation dataset was used to validate the model.

### 3.3 Severity evaluation using image segmentation techniques

In this article, images were divided into four disease severity levels: mild, moderate, severe, and profound. The intensity rates were finalized after discussion with domain expert. The categorization was performed according to the area of the foliage contaminated by paddy infection. If the infected area percentage was less than 25% then it was considered to be a mild infection. If the infected area ranged from 26% to 50% then it was considered to be of moderate severity. A leaf was considered to have a severe infection if the contaminated part of the plant was greater than 50% but less than 75% of the total leaf area. A profound level of infection severity was classified as an infected area greater than 75%.

#### 3.3.1 Severity evaluation

The evaluation of disease severity was based on the infected area of leaf owing to paddy diseases. The area of infection was calculated by employing the segmentation technique. Severity evaluation comprised leaf detection and then identification of the infected area of the leaf.

The whole process comprised five segmentation techniques: grayscale segmentation, threshold segmentation, edge detection, image masking, and histogram segmentation. In grayscale segmentation, according to their placements and gray values, each pixel in the medical grayscale image is translated into 3D coordinates as a pixel-features point cloud using the grayscale image segmentation method. Image thresholding is a straightforward but efficient technique for separating an image’s foreground from its background. By transforming grayscale photos into binary images, this image analysis technique is a type of image segmentation that isolates objects. Edge detection is a method of image processing used to locate areas in a digital image with sharp changes in brightness, i.e., discontinuities. The edges (or boundaries) of the image are those regions where the brightness of the image fluctuates dramatically. A smaller “image piece” is defined and used to alter a bigger image using the image processing technique known as masking. Many methods of image processing, such as edge detection, motion detection, and noise reduction, all start with the masking process. A grayscale value distribution known as an image histogram displays the frequency of occurrence of each gray-level value. The abscissa runs from 0 to 255 for an image size of 1,024 × 1,024 × 8 bits, and the total number of pixels is 1024 × 1024.

**Figure 4:**
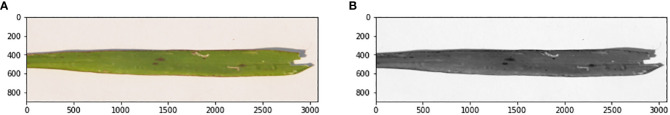
**(A)** Sample original leaf picture, **(B)** sample grayscale leaf picture.

First, noise (the background) is removed from the pre-processed image in the leaf detection phase. Then the detected leaf section of the image was used to calculate how much of the leaf was infected with paddy disease in the affected area detection phase. Leaf detection was performed using the grayscale, threshold, edge detection, and mask segmentation techniques. Histogram segmentation was used for the contaminated area detection in the leaf image. **Figure 4** shows the stepwise images of the severity evaluation. **Figure 4A** is the original leaf picture and **Figure 4B** is the image after applying the grayscale function.

**Figure 5:**
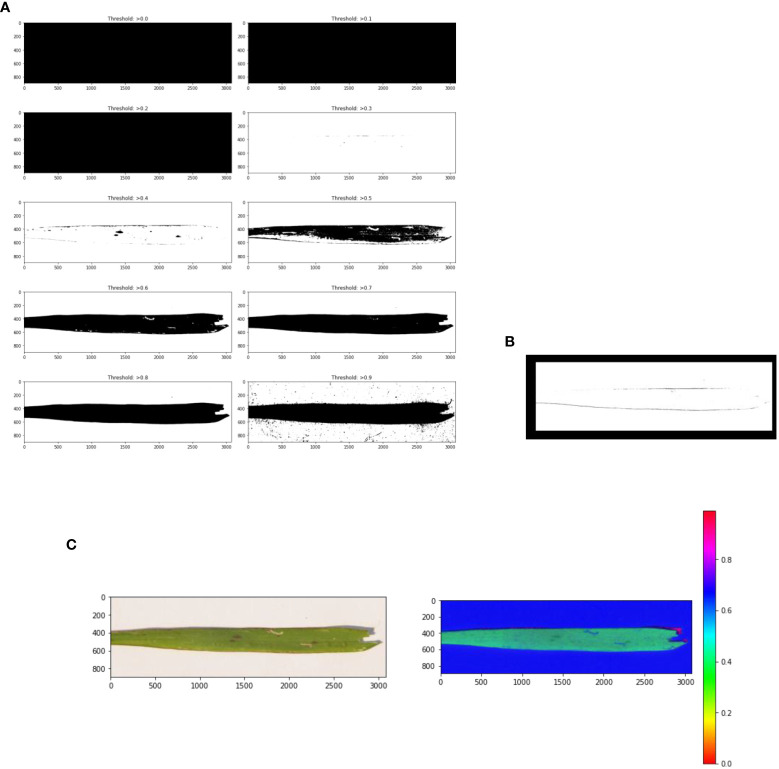
**(A)** Threshold images of the grayscale image at various threshold values, **(B)** leaf edge detection, and **(C)** image masking.


**Figure 6:**
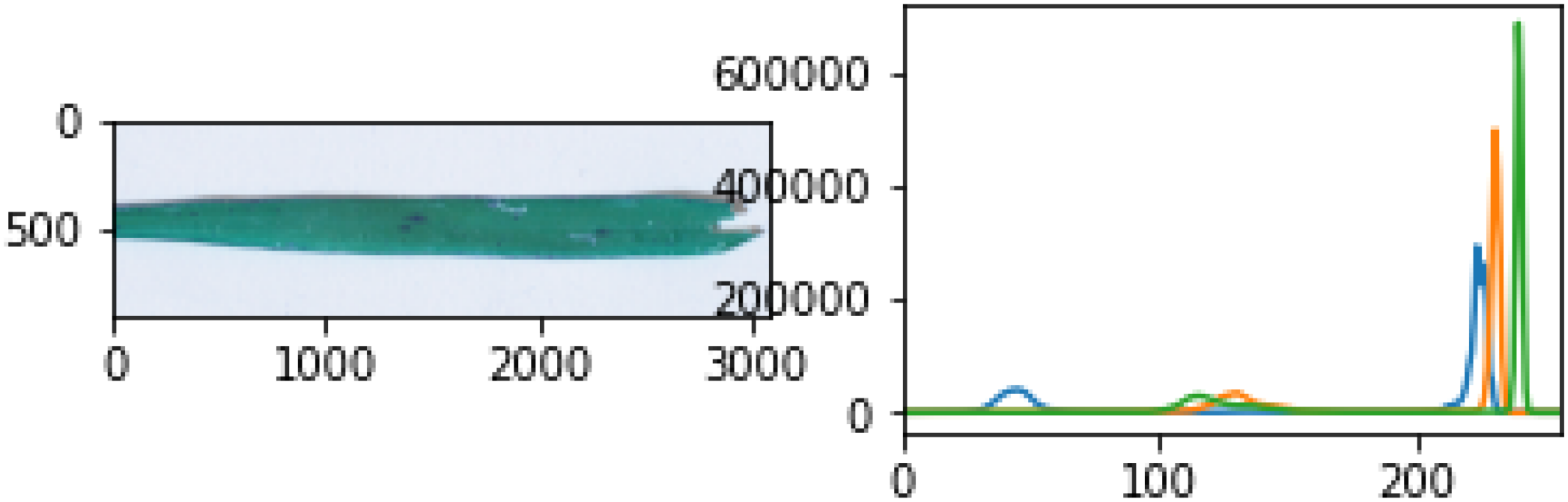
Image histogram to calculate disease severity.

The original image was first converted to a grayscale image to reduce the size of the image. Then various threshold values were applied to the grayscale image. **Figure 5A** shows an image at various threshold values. Leaf detection was performed using edge detection segmentation, which removes the background of the image. Various threshold values were applied and checked. After that, the histogram image provided the percentage of each color in the image. The yellow area of the leaf (as opposed to the green area) gave the infected area of the leaf using the formula:

(6) $$\text{Infected area }\%\;=\;\left({\text{P}}_{\text{yellow}}/{\text{P}}_{\text{total}}\right)\;\times \;100$$

where P is the number of pixels in the image, P_total_ is the number of pixels in the detected leaf, and P_yellow_ is the number of yellow pixels. **Figure 5B** shows the edge detection images. **Figure 5C** shows the mask images. **Figure 6** presents a histogram image with the percentage of each color.

#### 3.3.2 Severity annotation

Before the training process, the images were annotated. This was an essential step that helped the model to acquire the disease severity features. The precision of the annotation process strongly influences the training of the model. Given that multiple similar diseases can appear on leaves, knowledge of different diseases may support machine learning capabilities to classify diseases.

A horticultural scientist helped with the annotation of the images in the dataset. Experts determined the extent of damage to the plant, taking into account the various surface and shape parameters of the disease-affected part. The labels accounted for only external damage; this test did not account for internal damage. The annotated image’s output was presented as a bounding box and coordinates. Labeling the diseased regions on a picture was necessary for image annotation. After identifying and categorizing the degree of disease in a picture, Labeling, a freeware graphical visual annotation tool, recorded the information in an XML file with the proper xmin, xmax, ymin, and ymax values for each bounding box. The bounding box for each object was stored in an XML file. Working with annotation data that was stored in a different file for each image was challenging; therefore, each of these XML files was aggregated into a single CSV file using the Panda module. After that, the CSV file was divided into the four severity groups. The classification was based on the proportion of the leaflet where the bacterial infection is present. Then an object for each severity class was constructed. Then each line of image names and URLs in the object file was read iteratively. Object recognition accuracy was then measured for each object in each category.

### 3.4 Model generation

The proposed model is a fusion of two deep-learning classification techniques, CNN and SVM. The best characteristics of both algorithms were combined to improve classification accuracy. CNN was used for the feature extraction from the infected leaf images and SVM for the classification of the type of infection. A CNN-based model specifically created to categorize photos into various predetermined classes is known as an image classification CNN classifier. Accurate image classification is made possible by learning to extract pertinent features from input photos and map them to the appropriate classes. High-dimensional data, which are typical in many applications, such as text and picture categorization, can be handled well by SVMs. SVMs can effectively handle small datasets because the boundary needs to be defined by only a minimal number of support vectors. Python was used as the programming language to implement the model. The Jupyter Notebook platform was used for Python coding. Various computer vision libraries were used.

#### 3.4.1 Feature extraction

**Figure 7:**
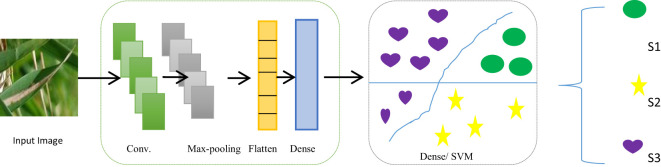
Structure of the proposed CNN–SVM hybrid classifier. Conv., convolution.

Two convolutional layers were used to extract features, accompanied by max-pool layers. **Figure 7** shows an exhaustive breakdown of the multi-class classification model’s structure. The model has seven levels in total. The input layer, which has a dimension of 64 × 64, is the top layer. Ninety-six filters of size 5 × 5 were then used to convolute input layer, creating a dimension of 32 × 32. The filter extracted features as it moved across the image. The output that includes details about the corners and edges of the image is called a feature map. These features were then processed by the maxpool layer, which has a filter of 2 × 2 magnitude and a stride worth of 2. The final image dimension is (16 × 16).

Two pairs of convolutional layers and a max-pooling layer were used in the suggested model. Sixty-four filters were used in the second convolutional layer of a 3 × 3 kernel size. The stride and filter size of the second pooling layer were both 2. Therefore, the image had the dimension of 8 × 8. The same padding and stride value of 2 was used for the rectified linear unit (ReLU) activation function throughout both convolutional layers. The output from each layer was passed on to the following layer, which uses it as its input. The flatten layer was then used to flatten the convoluted matrix (Adedoyin et al., 2022). The densely linked completely connected layer was then fed the output that had been flattened. **Table 4** lists each layer in the proposed model along with its parameters, kernel size, neurons, and output shape. It also lists the different levels of the GCS classifier’s activation functions. In addition, it displays the number of both trainable and total parameters.

**Table 4:** Detailed summary of Gan CNN and SV (GCS) model layers.

Layer	Layer type	Kernel size	Stride	Neuron size	Activation function	Output shape	Parameters (*n*)
conv2d_12 (Conv2D)	Convolutional layer C1	5 × 5	2	96 × 96	(Rectified Linear Unit) ReLU	Null, 32, 32, 96	7,296
max_pool2d_12 (MaxPool2D)	Max-pooling layer P1	2 × 2	2	96 × 96	–	Null, 16, 16, 96	0
conv2d_13(Conv2D)	Convolutional layer C2	3 × 3	2	64 × 64	(Rectified Linear Unit) ReLU	Null, 16, 16, 64	55,360
max_pool2d_13 (MaxPooling2D)	Max-pooling layer P2	2 × 2	2	64 × 64	–	Null, 8, 8, 64	0
flatten_6 (Flatten)	Flatten	–	–	–	–	Null, 4,096	0
dense_17	Sequential CNN	–	–	–	(Rectified Linear Unit) ReLU	Null, 288	1,179,936
dense_18	SVM	–	–	–	Softmax	Null, 3	867
Overall parameters: 1,243,458							
Trainable parameters: 1,243,458							

ReLU, Rectified Linear Unit.

#### 3.4.2 Classification

The SVM was used to classify paddy infections after the features have been pre-processed and extracted. The model was then flattened and fully connected layers were included. In the dense layer, the activation function was ReLU and used 288 units. In CNN, the SVM implementation takes place in the output layer. The L2 kernel was an activation regulator, and softmax was used on the output layer. The production layer was made up of three components, representing the total number of classes considered in the categorization problem. The classifier was then combined with the Adam optimizer, the squared hinge loss function, and accurateness as metrics.

SVM classifies images into just two groups because it is a binary classifier. However, this stage was where the precise degree of infection severity was assessed, and it involved a number of categories. For this, a regularizer was employed.

### 3.5 Train–test model

The hybrid model was then compiled, trained and verified using the training set and the test set, respectively.

#### 3.5.1 Training model

The classifier was compiled and trained with the training set. In this experiment, the model was trained with different numbers of epochs: 30, 50, and 100. The best results were found in 50 epochs. The model was validated against the validation dataset.

#### 3.5.2 Test model

**Figure 8:**
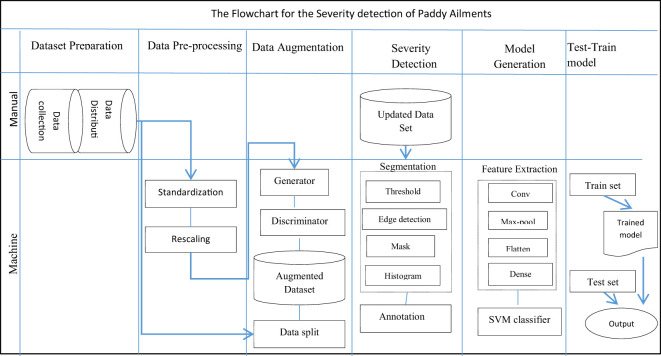
Detailed flowchart of the proposed multi-class hybrid classification model.

The trained model was then tested using the test set. A sample image was passed through the model and its ability to predict the correct paddy infection type and intensity was tested. **Figure 8** shows the structure of the complete severity and disease classification hybrid model with GAN augmentation. The actions in the classification model were categorized as manual or mechanical tasks. Then the sub-tasks were specified according to the phase and flow of the tasks. The dataset preparation task comprised data collection and data distribution, which was a completely manual task. Data pre-processing was carried out using the Python Keras library, which is integrated into the model itself, so this stage was performed by machine. GAN execution, segmentation, and classification model generation, were all automated tasks completed by the Python code.

### 3.6 Proposed algorithm

(1) Collect infected leaf images from primary sources (P_primary_) and secondary sources (P_secondary_). Dataset D = P_secondary_ + P_primary_. Secondary sources comprise Mendeley (D_mendeley_), GitHub (D_github_), kaggle (D_kaggle_), and UCI (D_uci_) datasets. P_secondary_ = D_uci_ + D_mendeley_ + D_github_ + D_kaggle_.

(2) Create a dataset folder for each category of paddy infection (D_bacterial blight_, D_blast_, and D_leaf smut_).

(3) Mount necessary libraries and datasets.

(4) Create the object for each category of disease.

(5) Perform pre-processing, which includes normalization and standardization.

(6) Perform GAN augmentation, which involves the generation of two models: the generator (GAN_generator_) and discriminator (GAN_discriminator_) models. The image is generated using the formula Img_GAN_ = GAN_generator_ × GAN_discriminator_.

(7) Save the augmented images in a separate folder.

(8) Merge the augmented images with the dataset according to the type of infection (D_bacterial blight_, D_blast_, and D_leaf smut_).

(9) Perform disease severity detection by applying image segmentation, threshold segmentation (S_threshold_), edge detection (S_edge_), masking (S_mask_), and histogram segmentation (S_histogram_) techniques to the infected image. Severity_label_ = f(S_histogram_, f(S_mask_, f(S_edge_, f(S_threshold_, image)))).

(10) Annotate the images with the severity labels (mild, moderate, severe, or profound).

(11) Repeat step 9 for each object, specifying the category of severity.

(12) Split the objects into a test–train set at a ratio of 20:80, D_test_:D_train_.

(13) Proposed model M_classifier_ = L_CNN_ ∪ L_SVM_.

(14) Extract features from the images using a convolutional neural network. The CNN comprises a pair of convolutional layers (L_conv_), a max-pooling layer (L_pool_), a flattened layer (L_flatten_), and a dense layer (L_dense_). M_CNN_ = 2× (L_conv_ ⊗ L_pool_) ∪ L_flatten_ ∪ L_dense_.

(15) Use the SVM layer in the CNN for the classification of image (L_SVM_).

(16) Compile and train the model with the training set (D_train_) using the formula M_train_ = δ(M_classifier_, D_train_).

(17) Test the trained model with the test set (D_test_) using the formula M_test_ = δ(M_train_, D_test_).

## 4 Results and discussion

The proportion of clearly specified data points in the set that is being trained is known as the training accuracy. Similar to resolution accuracy, validation accuracy describes the share of data samples that are correctly resolved from another sample. There were two sets in the dataset. The training images were in one set and the validation images were in the other set. Model training and validation were carried out using an 80–20 cross-validation procedure. Multiple mixed-image studies were performed for validation. The productivity of the classifier was tested using a new randomly selected image. The sparse categorical cross-entropy is used as loss function. The accuracy is 98.43% accomplished by the prototypical was 98.43%.

**Figure 9:**
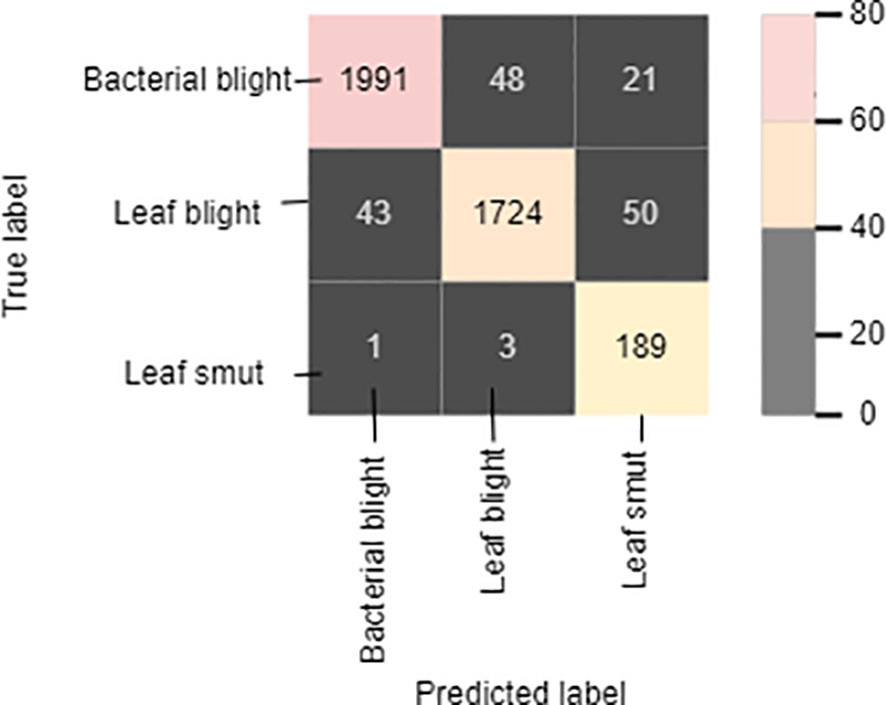
Confusion matrix.

The cross-entropy function of the classifier was optimized using the Adam optimizer. Cross-entropy loss is the most widely used function in deep-learning or machine-learning classification. It aids in evaluating a model’s accuracy in terms of 0s and 1s, from which we may later deduce the probability percentage. Out of the three diseases of the paddy, the model can identify leaf smut with an accuracy of 98.88%, precision value of 91%, recall of 97.7%, and F1-score of 94.23%. For bacterial blight disease, the model recorded an accuracy of 97.766%, precision value of 85%, recall of 84%, and F1-score of 84.5%. For the paddy blast disease, the model recorded an accuracy of 96.77%, precision value of 79%, recall of 84.75%, and F1-score of 81.77%. **Figure 9** shows the confusion matrix of the proposed model. The sample size used for the evaluation of the performance parameters was 20% of the images from each category of rice leaf disease.

The diagonally highlighted cells in the confusion matrix specify the number of images of bacterial blight, blast, and leaf smut that were predicted correctly. Other cells provide the number of images of the three rice diseases that were predicted incorrectly, meaning that the prediction was either a true negative or false positive. The proposed model correctly identified 1,991 images of bacterial blight from the dataset. In addition, 1,724 and 189 images of blast and leaf smut, respectively, were correctly identified. Cell (1,2) indicates that 48 images of blast were identified as bacterial blight. Similarly, three images of blast-infected leaves were identified as leaf smut by the proposed model, as shown in cell (3,2). In the case of cells (1,3) and (2,3), the model identified 21 and 50 images of bacterial blight and blast, respectively, as leaf smut-infected images. In cells (2,1) and (3,1), 43 images and 1 image of bacterial blight were wrongly identified as non blast and leaf smut, respectively.

**Figure 10:**
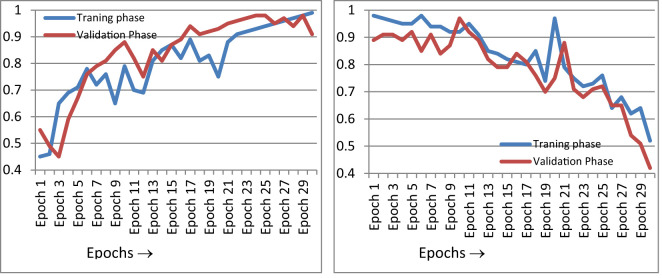
Performance curves. **(A)** Accuracy curve, **(B)** loss curve.

**Figure 10** gives the epoch-wise training and validation accuracy and loss value. **Figure 10A** shows an accuracy graph according to each epoch. The accuracy graph encompasses both the training and validation phase. **Figure 10A** shows that as the epoch increases, the accuracy of the fusion classifier’s prediction increases. This is because the model is being trained with each epoch. In **Figure 10B**, the loss curve is shown for the training and validation phase, according to each epoch. As the number of epochs increases and the classifier is trained, the loss function decreases. The precision of the proposed classifier for the classification of paddy disease type and severity increases as the epoch increases.

**Figure 11:**
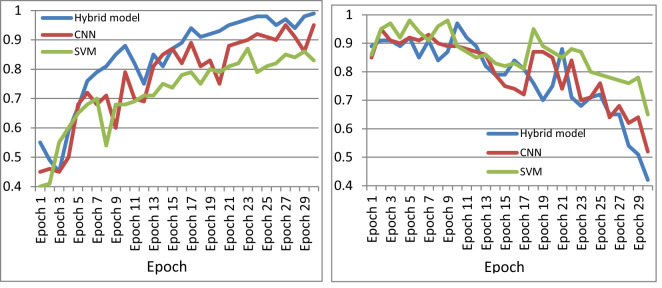
Comparison of performance of CNN, SVM, and the proposed model. **(A)** Accuracy curve, **(B)** loss curve.

The performance of the machine was further compared with existing cataloguing approaches using the same image set. **Figure 11** shows the accuracy curve and loss curve of various deep-learning classification models for the multi-classification of paddy diseases according to the type and severity of the disease when applied to the dataset created in this study. Two further algorithms were tested in this study: standard CNN and standard SVM for multi-classification. **Figure 11** indicates that the proposed hybrid model performed better than either individual approach.

**Figure 12:**
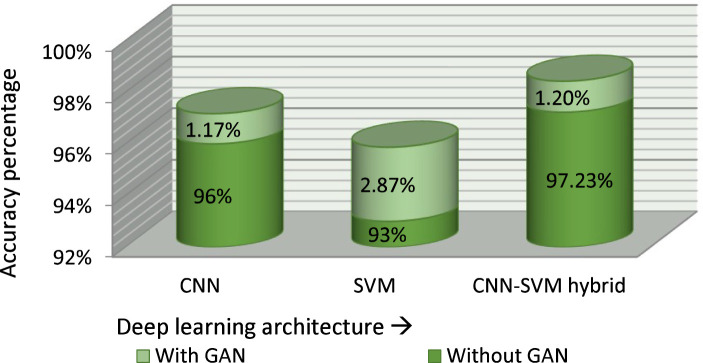
Effect of GAN on the model performance.

**Figure 12** shows the accuracy of various classification models with and without GAN augmentation. Four approaches were compared for accuracy with the proposed classifier model using the same dataset. The correctness achieved by the basic CNN classifier is 96% without GAN augmentation. GAN increased the accuracy of the basic CNN categorizer to 97.17%. A similar effect was seen on the standard SVM classification approach, with a GAN-augmented accuracy of 95.87% and an accuracy of 93% without GAN.

The performance of the proposed hybrid classifier was compared with the most prominent classifiers from the reviewed literature. **Table 5** shows the comparison of various approaches used for classification problems with the proposed hybrid model. The greatest accuracy attained with a CNN classifier was 99.45%, which was the best result out of all approaches. After CNN, a hybrid of CNN and SVM performed best with an accuracy of 99.20%. After that, the proposed model achieved an accuracy of 98.43%. A standard CNN with GAN augmentation achieved 98% accuracy in the classification problem. A Deep Convolutional Neural Network (DCNN) and SVM fusion model achieved 97.50% accuracy. A CNN combined with either the Internet of Things or VGG19 classifier attained an accuracy 95% and 95.40%, respectively, in classification problems. A 91% accuracy was achieved with a hybrid model of CNN and LSTM. CNN with transfer learning achieved 92.49% accuracy and random forest model attained 69% accuracy in classification problems.

**Table 5:** Performance comparison of the proposed approach with existing approaches used in classification problems.

Classification approach	Accuracy (%)
Proposed model	98.43
GAN–CNN	98
CNN with IoT	95
CNN	99.45
Random forest	69.00
CNN–SVM	99.20
CNN with transfer learning	92.49
Deep Convolutional Neural Network (DCNN) with SVM	97.50
CNN with LSTM	91.71
CNN with VGG19 model	95.40

IoT, internet of things; DCNN, Deep Convolutional Neural Network.

**Figure 13:**
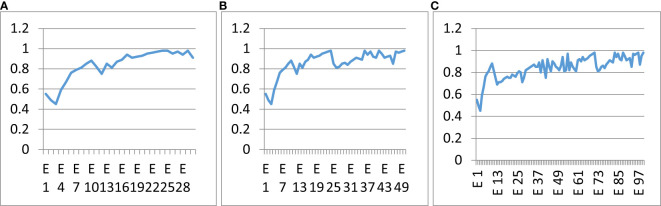
Influence of the number of epochs on the correctness of the anticipated model. **(A)** 30 epochs, **(B)** 50 epochs, **(C)** 100 epochs.

The proposed classifier was trained over a different number of epochs to study the influence of epochs on classification accuracy. **Figure 13** shows the accuracy curves of the proposed hybrid multi-class classifier at different numbers of epochs. **Figure 13A** shows the accuracy curve at 30 epochs, **Figure 13B** shows the accuracy curve at 50 epochs, and **Figure 13C** shows the accuracy curve at 100 epochs of training. There was no major difference in the precision and accuracy of the multi-class classifier at different epochs. The accuracy remained the same and had no major effect of epochs count. The optimal number of epochs for the training dataset is 50. GAN-augmented images from primary sources

**Figure 14:**
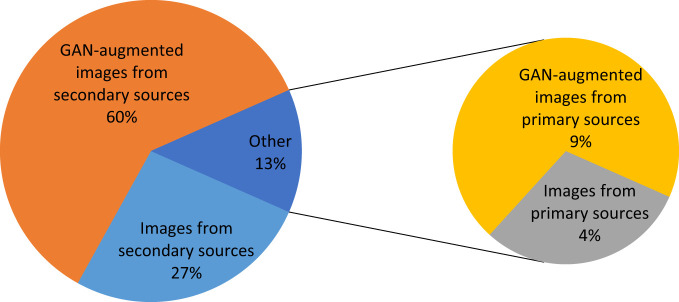
Effect of GAN augmentation on the size of the image set.

GAN has a major influence on the size of the dataset and hence on the accuracy of the model. **Figure 14** demonstrates the effect of GAN on the image set for the three paddy infections considered. As shown in **Figure 14**, 4% of the total dataset came from primary sources and 27% of the total dataset came from secondary sources. The GAN’s augmentation of the primary images constituted 9% of the total dataset and the GAN’s augmentation of the secondary images constituted 60% of the total dataset.

**Table 6** shows the results of the ablation study performed on the proposed model. Its shows that the accuracy of the proposed model is highest using the hybrid model and GAN augmentation. In machine learning, models have many different components, each of which affects the performance as a whole. Therefore, it is crucial to have a means of gauging how much these components contribute to the overall model. This is where the idea of an ablation study comes from, where specific components of the implementing model are removed to better understand the behavior. The proposed model consists of three components. In the ablation study, the effect of each component on the accuracy of the model was evaluated. The three components are GAN augmentation, feature extraction using CNN, and classification using SVM. The model’s accuracy was rated after training and testing using several GAN, CNN, and SVM combinations. The accuracy of the model without GAN augmentation and using SVM as a feature extractor and classifier while importing the same dataset was the lowest, at 93%. The accuracy of the model trained on the same dataset using CNN as the feature extractor and classifier and without GAN augmentation was 96%. Without GAN augmentation, the model using CNN as a feature extractor and SVM as a classifier achieved 97.2% accuracy. The CNN and SVM classifiers achieved 97% and 95% accuracy, respectively, with GAN augmentation.

**Table 6:** Results of the ablation study.

Model	Input		Deep-learning model		Accuracy
	Dataset	GAN augmentation	CNN	SVM	
Proposed model	✓	✓	✓	✓	0.9843
M1	✓	✗	✓	✓	0.9723
M2	✓	✓	✓	✗	0.9717
M3	✓	✓	✗	✓	0.9587
M4	✓	✗	✓	✗	0.96
M5	✓	✗	✗	✓	0.93

## 5 Conclusion and future applications

The biggest threat to agricultural progress is pathogenic diseases, which have a strong influence on overall production quality and quantity. As a result, a computer vision-based automatic diagnosis of rice leaf infections and the extent of infection is increasingly desirable in analytics. Deep-learning techniques, particularly CNNs and hybrid models with a CNN, have shown a promising ability to solve the difficulties in identifying infections. The combination of CNN and SVM was investigated to improve the ability to diagnose blight, blast, and leaf smut diseases in paddy leaves according to four disease severity levels. The image set comprised pictures of all three rice diseases and was compiled from both primary and secondary sources of data. A total of 533 images—202 images of bacterial blight, 218 images of rice blasts, and 113 images of leaf smut—were collected from primary sources. Four standard online repositories were used for secondary data collection. A total of 3,535 images—1,856, 1,599, and 80 images of bacterial blight, blast, and leaf smut infection, respectively—were collected from secondary sources. The dataset was then augmented using a GAN. The GAN increased the dataset from 4,068 images to 9,175 images. The augmented dataset was then pre-processed. Pre-processing comprised the standardization, normalization, and rescaling of the images. All these operations were implemented using the ImageDataAugmentor function of the Keras library. The severity level was then calculated using segmentation techniques. In this study, five segmentation techniques were used: grayscale, threshold segmentation, edge detection, masking, and histogram segmentation. The leaf detection process was accomplished using grayscale, threshold, edge detection, and mask segmentation techniques. The severity level was then annotated on the image. The severity evaluation was carried out using the pixel information from the histogram segmentation.

These images were then fed into the CNN–SVM fusion model for the categorization of the infection type. SVM was used as a classifier, and CNN was employed as a feature extractor. The test accuracy for blight, blast, and leaf smut disease on a sample of randomly chosen photos was 97%, 96%, and 98%, respectively. The results from the proposed hybrid multi-class classifier were compared with other approaches using the same dataset. When compared with supplementary algorithms tested on the same image set, the proposed model yielded the best results. The approaches used for the comparison were standard CNN, standard SVM, standard SVM with GAN, standard CNN with GAN, and CNN–SVM without GAN, and their respective accuracies were 96%, 93%, 95.87%, 97.17%, and 97.23%. To increase its size, 69% of the dataset was generated using GAN augmentation techniques. Secondary sources constituted 27% of the dataset, and primary sources constituted 9% of the dataset. The proposed model helps in the identification of rice leaf disease and the level of disease severity, which can help farmers to apply the appropriate remedies to stop the spread of the disease to other healthy plants. The identification of disease and determination of an exact severity level also enables farmers to predict the degree of loss of crop productivity.

In the future, this methodology can be utilized for the multi-categorization of other plant infections for the same or different crops. The proposed model works with various other datasets for a variety of crops. The proposed approach is useful for predicting the crop yield of a field based on losses due to various crop infections.

A limitation of this study is that the dataset used contained images showing only sections of infected plant leaves. A better method would be to choose only images showing complete leaves from the infected plant for the calculation of disease severity.

## Data availability statement

The original contributions presented in the study are included in the article/supplementary material. Further inquiries can be directed to the corresponding authors.

## Author contributions

Conceptualization: SL, VK, JR, AB. Methodology: SL, VK, JR, AB, TG. Formal analysis and investigation: SL, VK, JR, TG, JK, AB, DG, SS. Writing—review and editing: SL, VK, JR, TG, JK, AB, DG, SS. All authors contributed to the article and approved the submitted version.
